# Surviving salt fluctuations: stress and recovery in *Halobacterium salinarum*, an extreme halophilic Archaeon

**DOI:** 10.1038/s41598-020-59681-1

**Published:** 2020-02-24

**Authors:** P. Vauclare, F. Natali, J. P. Kleman, G. Zaccai, B. Franzetti

**Affiliations:** 1 University Grenoble Alpes, CNRS, CEA, IBS, F-38000 Grenoble, France; 2 CNR-IOM, OGG, F-38042 Grenoble, France; 3 Institut Laue Langevin (ILL), F-38042 Grenoble, France

**Keywords:** Biophysics, Microbiology, Ecology

## Abstract

Halophilic proteins subjected to below about 15% salt *in vitro* denature through misfolding, aggregation and/or precipitation. Halobacteria, however, have been detected in environments of fluctuating salinity such as coastal salterns and even around fresh water springs in the depths of the Dead Sea. In order to identify the underlying mechanisms of low salt survival, we explored the reactivation capacity of *Halobacterium (Hbt) salinarum* sub-populations after incubation in low salt media and recovery in physiological salt. Respiratory oxygen consumption was assessed in stressed cells and cell viability was estimated by Live/Dead staining and flow cytometry. *In vivo* neutron scattering experiments showed that the recovery of *Hbt salinarum* sub-populations exposed to severe low salt conditions is related to a rapid retrieval of functional molecular dynamics in the proteome. In the hypothesis that the observations on *Hbt salinarum* have wider relevance, they could be of key ecological significance for the dispersion of extremophiles when environmental fluctuations become severe.

## Introduction

Halophilic Archaea require hypersaline conditions (up to 5 M NaCl) for optimal growth conditions found in various habitats on Earth, such as natural hypersaline lakes^1^, salterns or subterranean salt deposits^2^. Extreme halophilic Archaea like *Halobacterium salinarum* are of special interest because they resist the external osmotic pressure mainly by the accumulation of correspondingly high intracellular KCl concentrations^3^. Cellular accumulation of K^+^, Cl^−^, and exclusion of Na^+^ requires energy dependent mechanisms achieved by Na^+^/H^+^ antiporter systems and K^+^ transport systems^4^. It was baffling to the scientific community that essential macromolecular interactions were not inhibited in the close to saturated salt cytosol of extreme halophiles and, furthermore, that salt concentration bellow 2 M in which mesophilic microorganisms thrive resulted in halophilic enzyme inactivation^5–7^. A resolution to the puzzle was proposed from *in vitro* experiments on model enzymes, which revealed the requirement for high salt concentration to stabilize halophilic protein structures through solvation shells made up of hydrated salt ions^8^. The property is correlated with negatively charged protein surfaces, via an enrichment in acidic amino acids and marginal hydrophobic amino acids that favor repulsive interparticle forces to avoid aggregation in the high salt environment^9,10^. Site-directed mutagenesis of a halophilic protein from *Haloferax volcanii* and its mesophilic homologue and the subsequent NMR and thermodynamics characterizations indicated that surface aspartic and glutamic acids allow reducing the interaction surface between the protein and the solvent, which is beneficial in low water activity conditions to maintain an hydration shell^11^. Neutron scattering measurements of the molecular dynamic properties of the MalDH enzyme from *Haloarcula. marismortui* and of the native proteome from *Hbt. salinarum*^12–16^ indicated that halophilic proteins display specific biophysical adaptation properties to high salt reflected by higher structural resilience, compared to mesophilic systems. It is likely, therefore, that most halophilic proteins display an obligatory requirement for high salt conditions in order to be active and stable. As a consequence, it is expected that intracellular ionic fluctuations in response to external concentration differences pose non-trivial problems for cellular biochemistry in extreme halophiles.

In coastal salterns extreme halophiles populations can be exposed to seasonal fluctuation in salt concentration, fresh water supply as well as transient rains and flooding episodes. Halophilic organisms exhibit a range of stress responses to counterbalance the deleterious effects induced by low salt. *Hbt. salinarum* accumulates a heat shock protein in the cytosol (the thermosome, known to stabilize proteins during thermal stress) as well as low salt stress specific chaperonin complex called Ssp45^17^. Proteasome complexes, involved in the clearing of misfolded or damaged proteins are also induced by moderate hyposaline stress (~2.5 M NaCl)^18^. Other responses include down-regulation of the translational apparatus and modulation of the expression of genes encoding for enzymes associated with the primary metabolism^19,20^. Furthermore, the expression of many genes encoding for peptides and ion transporters, such as those that regulate K^+^ and Na^+^ concentration in the cytosol, are salt- dependent^21,22^.

*In vitro*, halophilic proteins subjected to low salt denature through misfolding, aggregation and/or precipitation. In its natural environment, however, *Hbt salinarum* is exposed to large salt fluctuations. In this context, we explored the reactivation capacity of Hs sub-populations after incubation in low salt media, 2.5 M down to 0.5 M NaCl, and recovery in physiological salt (4.2 M NaCl). Respiratory oxygen consumption was assessed in stressed cells and cell viability was estimated by Live/Dead staining and flow cytometry. The molecular dynamics of the proteome represents a good indicator of protein functionality. *In vivo* neutron scattering experiments showed that the recovery of *Hbt salinarum* sub-populations exposed to severe low salt conditions is related to a rapid retrieval of functional molecular dynamics. The observations are of key ecological relevance in the context of climate change and the dispersion of extremophiles in fluctuating environments.

Also, Dawson *et al*.^23^ observed that halophilic archaea regulate the proportion of unsaturated diacylglycerol glycerol diethers (DGDs) in the membrane lipids according to salt fluctuations in the environment.

Compatible solutes may also exist in extreme halophilic Archaea as suggested by comparative genomic analyses^24^ but these organisms display a limited capacity to adapt to salt concentration below their optimum^4^. In the case of *Hbt. salinarum* we have shown that below 2 M NaCl (a value which induces unfolding of the model *Haloarcula marismortui* enzyme, MalDH, in absence of cofactors) halophilic cells suffer a high mortality rate^15^. In the same study, molecular dynamics parameters of the proteome were measured *in vivo* by neutron scattering for cultures exposed to various lower salt concentrations. Already at 2.5 M salt, significant protein molecular dynamics perturbations were observed, similar to those in heat stressed *Hbt. salinarum* cells^14^. These observations suggested that alteration in cytosolic protein solubility/stability represent a major cause of cellular stress in low salt conditions. It is assumed that such modification would cause irreversible damage, thus preventing cellular reactivation when optimal environmental hypersalinity is restored. Considering the stress effect of low salt condition on halophilic systems, it is surprising that several studies highlighted the presence of halophilic Archaea in low salt conditions in soils, human or plant microbiomes and in the stratosphere^25–29^. In this work, we explored the reactivation capacity of *Hbt. salinarum* sub-populations after incubation in low salt conditions. We studied different cell properties after salt shock (2.5 M down to 0.5 M NaCl) and recovery in high salt (4.2 M NaCl). Cell viability was estimated by Live/Dead staining and flow cytometry, which allowed us to follow in real time multiple cellular parameters including cell membrane integrity. Because *Hbt. salinarum* is an aerobic heterotroph, we also assessed the respiratory oxygen consumption of stressed cells. *Hbt. salinarum* proteome molecular dynamics under different conditions was then measured *in vivo* by neutron scattering. In previous work, effects of the environment on protein dynamics and its direct relevance for biological activity^30^ was illustrated by neutron scattering studies of proteome molecular dynamics, *in vivo*, on bacterial cells adapted to different physiological temperatures^31^. In the study, the mean structural resilience of the proteome, expressed as an effective force constant, was found to be adapted in order to maintain flexibility appropriate for activity at the physiological temperature. Thus, psychrophiles had ‘softer’ structures, flexible at low temperatures, while the thermophile proteome was ‘harder’ to maintain stability at high temperatures. The measure of flexibility converged to a constant value at physiological temperature for all the organisms examined. Similarly, the current study established that molecular dynamics in *Hbt. salinarum* is adapted to physiological high salt and constitutes a sensitive measure of cellular distress at the molecular level when the salt concentration drops. Here, the neutron scattering study revealed that, in the cytosol of reactivable cells, the necessary condition of functional molecular dynamics was rapidly restored after being significantly affected by low salt stress. With this approach, we obtained evidence that sub-populations of *Hbt. salinarum* can survive severe low salt conditions to start multiplying again when the salt concentration rises back to physiological values. These observations may have general ecological significance in the context of dispersion of extremophiles in fluctuating environments.

## Results

### *Hbt. salinarum* cell growth during low salt stress and re-inoculation in high salt optimal conditions

**Figure 1:**
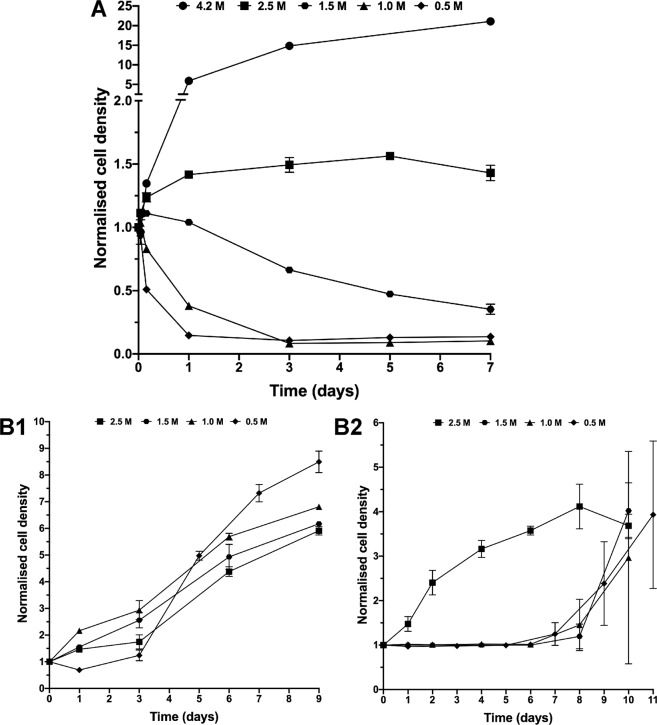
(**A**) Normalized cell density of *Halobacterium salinarum* as a function of time for different salt (NaCl) concentrations. Cells grown in 4.2 M NaCl were harvested then cultivated at 37 °C under aerobic conditions in rich medium with 2.5 M, 1.5 M, 1.0 M and 0.5 M NaCl. (**B**) Growth kinetics of *Hbt. salinarum* during recovery. Cells were shocked in 2.5 M (■), 1.5 M (▲), 1.0 M (●) and 0.5 M (x) NaCl for 1 hour (**B1**) or 7 days (**B2**) and then were incubated in 4.2 M NaCl for recovery. Growth was monitored as optical density of the culture. Each experiment was carried out in triplicate. Errors bars represent standard errors.

In extreme halophiles, low salt conditions elicits the induction of stress response systems suggesting that part of the halophilic population could tolerate significant diminutions of environmental salt conditions. To test this hypothesis, *Hbt. salinarum* cultures were diluted in growth media containing different NaCl concentrations and growth was measured for 7 days. Cells exposed to 2.5 M NaCl maintained their ability to divide for a few days (Fig. 1). The growth rates, however, were up to 5 to 8 times lower than for control cells in 4.2 M NaCl. Growth then slowed down progressively, suggesting that cells entered a phase similar to a stationary phase. The stressful effect of low salt concentration appeared to be particularly pronounced at concentrations lower than 1.5 M NaCl (Fig. 1A). Below 1.5 M, growth curves exhibit a typical logarithmic biphasic killing pattern described in many bacteria exposed to harmful environmental stress^32^, which may reflect an initial exponential phase corresponding to a sensitive population and a second phase with a much slower decline curve follow by a plateau. Interestingly, we observed that all stressed cells inoculated into hypersaline physiological medium switch back to a normal growing cell population after a given time period (Fig. 1B), suggesting that recovered cells, including those that have undergone extreme low-salt shock, possess the capacity to multiply again. Cells incubated in 2.5 M started to grow instantaneously. A lag phase of three days was recorded for cells incubated during 1 hour in 0.5 M NaCl (Fig. 1B1) and of 7 days for cells incubated during one week in 1.5 M, 1 M or 0.5 M NaCl (Fig. 1B2). The observations suggest that part of the low salt stressed cells can undergo a reactivation process when they encounter optimal saline conditions.

### Flow cytometry analysis of *Hbt. salinarum* cells structural integrity under low salt stress and high salt reactivation

The viability of stressed *Hbt. salinarum* cells was assessed by flow cytometry analysis^33^. Mainly used for eukaryote and bacterial analysis, the extreme physico-chemical conditions around *Hbt. salinarum* were limiting for the application of flow cytometry. The method was tested for the first time on extreme halophiles in this study and we confirmed its effectiveness by using SYTO 9/Propidium iodide staining, a powerful indicator of membrane integrity (LIVE/DEAD™ Baclight™ bacterial viability kit, Molecular Probes)^14^. Propidium iodide (PI) fluorescent nucleic acid stain identifies damaged cells that lost membrane integrity (PI+) (especially significant considering the key role of the membrane in bioenergetics and maintenance of the electrochemical potential of K^+^, Na^+^, and H^+^ ions^33^), while SYTO9 DNA labeling distinguishes cells from debris, empty cell ghosts, and DNA-damaged subpopulations (Syto−).

**Figure 2:**
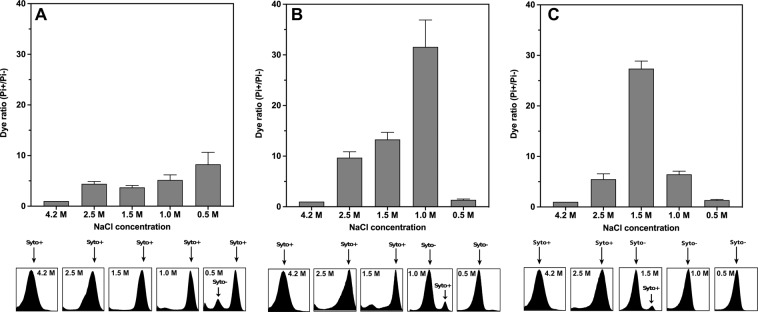
Flow cytometry analysis of membrane integrity (PI labeling) and DNA staining (Syto labeling) of *Hbt. salinarum* cells. The cells were incubated for 1 hour (**A**) and 24 hours (**B**) and 7 days (**C**) in standard growth medium (4.2 M NaCl, control) or in low NaCl conditions (2.5 M, 1.5 M, 1.0 M and 0.5 M At the top, PI+ (membrane integrity) cell populations detected at 645 nm (PI). At the bottom, histograms of Syto9 (DNA labelling) staining showing changes in the fluorescence intensity (at 528 nm) of cells exposed to various salt concentrations. Damaged (Syto−) and undamaged DNA (Syto+) populations are indicated by arrows. Each experiment was carried out in triplicate. Errors bars represent standard errors.

Aspects of different cell populations are shown in scanning and transmission electron micrographs in (Fig. S1). The cell populations were analyzed after 1 hour (Fig. 2A) and 24 hours (Fig. 2B) incubation at 37 °C under agitation in growth media containing 4.2 (control), 2.5, 1.5, 1.0 and 0.5 M NaCl, respectively. As expected, the accumulation of PI+/PI−, Syto− and lytic cells increased proportionally to the diminution of environmental salt concentration and stress duration. After moderate salt stress (2.5 M), however, a significant part of the initial population remained intact, even after 7 days (Fig. 2C). It is noteworthy that after 1 hour in 0.5 M NaCl a significant proportion of the cells retained structural and DNA integrity (Fig. 2A). After a prolonged incubation in low salt concentrations (24 hours and 7 days), the majority of the harvested cells displayed considerable DNA damage and membrane injury (Fig. 2B,C). However, for cells incubated in NaCl 0.5 M for 24 hours or in NaCl 1 M and 0.5 M for 7 days, the (Pi+/Pi−) decreased significantly to reach values close to that of unstressed cells. A possible explanation for these results is that most cells are lysed under these extreme conditions, and the signal is dominated by a small fraction of surviving cells.

**Figure 3:**
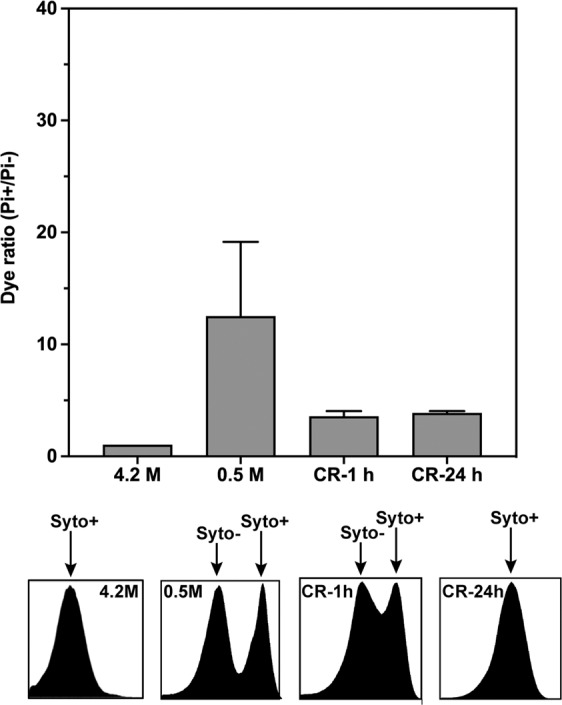
Flow cytometry analysis of membrane integrity (PI labeling) and DNA staining (Syto labeling) of *Hbt. salinarum* recovered cells. The cells were incubated in 4.2 M NaCl (control) or after low salt shock (0.5 M-1h) for one hour followed by cell recovery in 4.2 M NaCl for 1 hour (CR-1h) and 24 hours (CR-24h). At the top, PI+ (membrane integrity) cell populations detected at 645 nm (PI). At the bottom, histograms of Syto9 (DNA labelling) staining showing changes in the fluorescence intensity (at 528 nm) of cells exposed to various salt concentrations. Damaged (Syto−) and undamaged DNA (Syto+) populations are indicated by arrows. Each experiment was carried out in triplicate. Errors bars represent standard errors.

To monitor reactivation, low salt (0.5 M NaCl) stressed *Hbt. salinarum* samples were subsequently analyzed after harvesting and incubation for 1 and 24 hours, respectively, back in physiological saline medium (Fig. 3). After 1 hour incubation (CR-1h), the (Pi+/Pi−) ratio decreased indicating less membrane damage compared to the initial stressed cells; on the other hand, the Syto− and Syto+ pattern while similar to that of the stressed cells (Fig. 2A) shows a gradual shift towards Syto+ values indicating fewer cells with DNA damage. The tendency is confirmed in CR-24h, in which only one population is observed with undamaged DNA and intact membranes. Taken together, these results indicated that a significant sub-population of *Hbt. salinarum* cells can maintain its membrane integrity and is competent to recover from low salt stress.

### Time-lapse microscopy of low salt stressed *Hbt salinarum* cell populations during high salt reactivation

**Figure 4:**
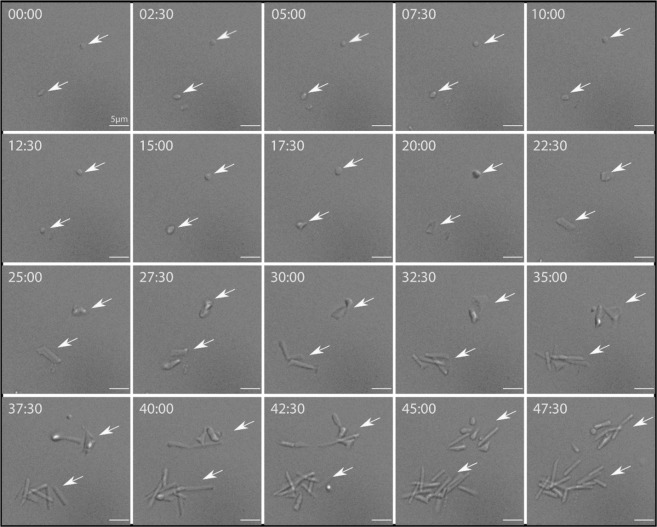
Time-lapse light microscopy. *Hbt. salinarum* morphological changes were monitored during recovery in 4.2 M NaCl growth medium after a low-salt shock (1 h) at 0.5 M. Cells were grown at 37 °C in the microscopy chamber and observed by Dichroic Interference Contrast. Images were collected every 30 minutes over a period of 48 hours.

In an exploration of *Hbt. salinarum* reactivation, we examined the evolution of cell morphology by time-lapse microscopy (Dichroic Interference Contrast imaging; DIC) during recovery in 4.2 M NaCl following incubation in 0.5 M NaCl (Fig. 4) and 2.0 M NaCl (Fig. S2) growth medium for 1 hour. Stressed cells (0.5 M NaCl) exhibited a round shape about 1 µm in diameter during the first 16 hours of culture in hypersaline conditions. At about 32-36 hours small islets of cells regaining the typical rod-like shape of *Hbt. salinarum* asynchronously appeared. Interestingly, rod-shaped cell formation did not follow classical binary fission, used by most Archaea for cell division. Cell division rather initiated from flat sacculi that eventually acquired conventional rod morphology. The rod-shape cells then elongated and split into two daughter cells. The cycle was repeated as long as cells were cultured under favorable conditions, consistent with growth rates presented in Fig. 1.

### Assessment of the metabolic state of stressed and reactivated *H. salinarum* populations

**Figure 5:**
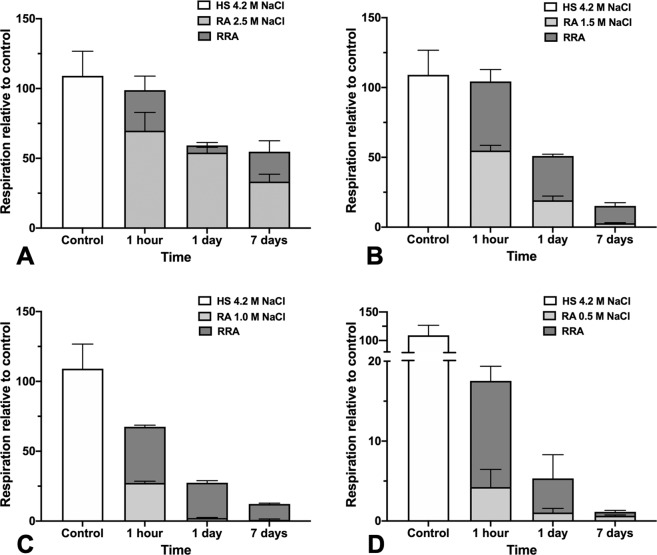
Respiration rate of *Hbt. salinarum*. Respiration activity (RA, light grey) of cells in low-salt shock (2.5 M, 1.5 M, 1 M and 0.5 M NaCl) measured at 1 hour, 24 hours and 7 days and recovery respiration activity (RRA, dark grey) immediately after transfer of stressed cells in standard 4.2 M NaCl medium. Oxygen consumption of cells was determined at 37 °C using a Unisense oxygen sensor. The experiment starts with basal respiration of unstressed cells (HS 4.2). Harvested cells were then exposed to various low-salt concentrations ((**A**) 2.5 M NaCl, (**B**) 1.5 M NaCl, (**C**) 1.0 M NaCl and (**D**) 0.5 M NaCl) during different periods of time for respiration measurement. Finally, oxygen consumption of recovered cells was measured immediately after incubation of stressed cells in 4.2 M NaCl. Basal respiration of unstressed cells was set to 100% in each case. Results are mean values for three independent experiments. Time on the x-axis corresponds to the low-salt incubation time. Note that different scales were used in figure (**D**).

The effect of low salt exposure on respiration is shown in Fig. 5 (light grey bars). We observed that the respiratory sensitivity of Haloarchaea is NaCl concentration and time-dependent. As illustrated in Fig. 5, lower salt concentration led to a loss of respiratory rate; a phenomenon amplified when the incubation time increased. The longer the shock, the higher the minimum value of salt concentration for which respiration rate reached zero. The O_2_-uptake rate of stressed cells decreased notably for cells exposed to below 1.5 M NaCl for 24 hours or 7 days. The progressive loss of respiration, under these conditions, parallels slow or stopped *Hbt. salinarum* growth in the same conditions (Fig. 1). Figure 5 also shows the respiration activity of low salt stressed *Hbt. salinarum* cells incubated back in a high salt medium (dark grey bars). It shows that the respiratory activity increases rapidly after transfer to 4.2 M NaCl physiological medium (Fig. 5, RRA), even for cells exposed for a long time to extreme low salt concentration (Fig. 5C,D). In all cases, respiration increases instantaneously including for the longest shock (7 days) at the lowest salt concentration (0.5 M NaCl). This cannot be the result of undesired contaminations, as demonstrated by control measurements of the recovery medium alone (data not shown). The observations suggested that a significant part of the cell population is still able to produce energy even during a brutal salt shock or when exposed to prolonged periods of stress at moderately low salt concentrations.

### ***In vivo*** molecular dynamics parameters in low salt stressed and high salt reactivated samples

As discussed previously, the molecular dynamic state of the cellular proteome represents a robust indicator of cellular fitness^14,15,31^. Macromolecular denaturation through unfolding leads to lower effective force constants indicating less rigid structures. According to *in vitro* experiments on a model halophilic protein and to our previous work on *Hbt. salinarum* cells exposed to moderate salt stress (2 M NaCl)^15^, a drop in intracellular K concentration should induce important damage to the proteome. Here, we performed *in vivo* neutron scattering experiments to characterize the molecular dynamics parameters of the *Hbt. salinarum* proteome exposed to low salt.

**Figure 6:**
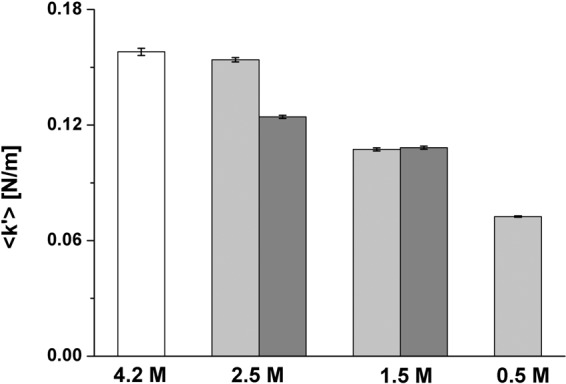
Mean effective force constant <k’> of *Hbt. salinarum*, for different conditions. Columns represent values for a given salinity and incubation time (1 hour, light grey or 24 hours, dark grey). The white column is the control sample at physiological salt molarity. Errors bars represent standard errors.

**Figure 7:**
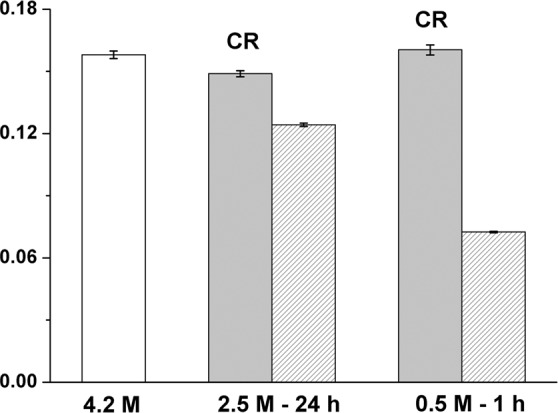
Mean effective force constant <k’> of *Hbt. salinarum*. The reactivated samples (CR, solid grey blocks) are compared to the corresponding value under stress (dashed patterns) and the control (white column). Errors bars represent standard errors.

Samples were measured on the IN13 spectrometer at the ILL (8 µeV energy resolution sampling a 0.1 ns time scale), as described in Vauclare *et al*.^15^. Sample preparation for the neutron experiments is described in Materials and Methods. A centrifugation step prior to sample load in the measurement cell eliminated most of cell debris and lysed material. We considered, therefore, that the measured signal was dominated by the fraction of intact cells in the low salt stress samples to provide relevant information on their functional state. The experiments displayed in Fig. 6 correspond to *Hbt. salinarum* cultures exposed to 2.5 M and 0.5 M NaCl concentrations for 1 hour or 24 hours. The effective average force constant <k’> (N/m) stabilizing macromolecular structures within the cell was calculated from the temperature dependence of mean square displacements (MSD). The <k’> value fell progressively with lower salt concentrations and longer exposure times, indicating a softening of the resilience of macromolecular structures, as observed previously for heat-stressed cells and cells exposed to a moderate low salt stress for 1 h^14,15^. The scattering vector modulus (Q) range of IN13 extends from 0.3 Å^−1^ to almost 5 Å^−1^, corresponding to a length scale from a few ångströms (at low Q) to a fraction of an ångström. The analysis was limited to the low Q end, where the time-length window is appropriate for MSD of macromolecular internal motions as well as the larger fluctuation amplitudes involved in unfolding processes (Fig. S3). Unfolded proteins have been measured by neutron scattering to display lower resilience than folded states^34^ so that the effective force constants in the low salt samples in Fig. 6 indicated less rigid macromolecular structures, reflecting perturbation in the folding state of a significant part of the *Hbt. salinarum* proteome. Even if the unfolded structures tended to aggregate, it is not unlikely on the time scale of internal motions that such aggregates would be less resilient than the compact cores of native states^34^. We subsequently measured the effective average force constants of *Hbt. salinarum* after 1 hour and 24 hours of high salt recovery (Fig. 7). The 2.5 M NaCl and 0.5 M NaCl concentrations, with further incubation at 4.2 M NaCl for 1 hour, were chosen to represent a moderate and an extreme stress conditions. The <k’> values for the low salt stressed samples are given by the dashed blocks (data from Fig. 6). The <k’> values of the recovered samples (solid grey blocks) have themselves recovered the physiological value of the 4.2 M NaCl control (white block), indicating that a major part of the cells that resisted the low salt conditions had rapidly regained a favorable internal macromolecular environment, a necessary precondition for the recovery of membrane integrity, respiration and viability.

## Discussion

Low salt concentrations, *in vitro*, were found to be strongly destabilizing for model halophilic proteins^8,10^. Previous neutron scattering experiments established this was also the case in the cytosol of *Hbt. salinarum* exposed to moderate low salt^15^. In this work, we extended the neutron scattering observations to extreme low salt shock. And, as expected, a more substantial dynamic perturbation of the proteome was observed. Furthermore, we characterized cellular and physiological modifications resulting from low salt stress and shock. *Hbt. salinarum* exposed to low salt stops growing. Most cells adopt a spherical shape; an increasing fraction of dead cells and cells with damaged membranes is detected together with a dramatic decrease in residual respiratory activity. Strikingly, even for the sample exposed to 0.5 M NaCl, we observed the return of respiration and proteome molecular dynamics was rapidly restored to control values already after incubation for 1 hour back in 4.2 M NaCl. The evolution of cell architecture suggested that cell division property is recovered after several hours incubation in 4.2 M NaCl. Round-shaped cells with a dense cytosol progressed to undefined flat saccules; after about 32 to 36 hours, small islands of rod-shaped cells, typical of *Hbt. salinarum*, appeared asynchronously and continued to divide. This cycle was repeated, consistent with the growth rate measurements. The observations indicated that despite the harsh low salt shock exposure, which significantly affected the molecular dynamics of the proteome and consequently cell metabolism, a fraction of cells maintained their capacity to recover. Different non-exclusive hypotheses could explain the observation: (1) large intracellular molecular assemblies, like chaperonins, proteasomes, peptidases, that were shown to be more resistant to low salt conditions *in vitro*^17,18,35^ trigger the recovery; (2) low salt conditions induce the accumulation of protective stress response molecules, such as chaperones or compatible solutes; (3) The formation of persisters, dormant stress-tolerant phenotypic variants first identified while investigating antibiotic resistance in bacteria^36^. Even though the latter hypothesis is supported by the biphasic time-kill curves, further investigation will be required to establish whether or not *H. salinarum* low salt stress survivors share other properties specific to persisters^37^.

The neutron scattering results showed that the presence of cells that could be reactivated in low salt stress populations (2.5 M and 0.5 M NaCl) was not due to functional molecular dynamics parameters being maintained during the stress event. This implies that a modified molecular dynamics landscape, reduced metabolic activity and morphological changes (in the case of the 2.5 M sample) did not prevent the recovery of *Hbt. salinarum* cells from reactivating even if the perturbation were maintained for long periods, and that the reactivation process correlated with the restoration of functional molecular dynamic parameters. The phenomenon can be rapid in the case of short stress, consistent with the salt-dependent halophilic protein solvation/activation hypothesis^7^. The rapid recovery, after incubation in physiological salt, of proteome molecular dynamics and respiration indicates that the perturbation is reversible. Recovery could be due to the restoration of the favorable halophilic protein-solvent interactions at high salt and/or to de novo synthesis of key enzymes by the translation machinery, both processes fueled by intact membrane ion pumps and respiratory complexes. Note that other cellular functions may be much slower to appear as suggested by the later onset of cell division.

In this work, we highlighted the capacity of specific *Hbt. salinarum* cells to overcome the physico-chemical damage of low salt on halophilic protein molecular dynamics in order to recover when put back in physiological medium. The observation contributes to the understanding of how halophilic Archaea, exposed to important salt fluctuations occurring in their natural environments, can survive and disseminate in low salt conditions, such as in soils, human or plant microbiomes and in the stratosphere, where they have been shown to occur^25–29^. The characterization of in cellular molecular dynamics of stressed and reactivated cells illustrated the physico-chemical aspects of the survival process. The determination of the cell biology mechanisms involved in the selection and reactivation of resistant cells is a major challenge to be tackled in the future, in particular by combining single cell metabolomics and proteomics.

## Materials and Methods

### ***Hbt. salinarum*cells: cultivation and growth conditions**

*Halobacterium salinarum* R1 (DSM 671) is the hyperhalophilic archaea strain used for the study. Cells were grown at 37 °C, with shaking at 110 rpm in standard hypersaline medium containing 4.2 M NaCl, 160 mM MgSO_4_, 10 mM Trisodium citrate and 26 mM KCl (Oesterhelt and Stoeckenius 1974) under aerobic conditions. For salt stress shock experiment, cells were grown to the mid exponential phase (OD 600 ~ 0.5–0.8) then centrifuged at ~4000 × g. For low salt stress experiments, the cell pellets were suspended in standard cultivation medium containing the desired NaCl concentration and cells were further cultivated for one hour, 24 hours and 7 days at 37 °C under agitation. For the reactivation process, stressed cells were pelleted again and re-suspended in standard hypersaline media and cultivated at 37 °C with shaking. The impact of the remaining salt concentration in the cellular paste on the final NaCl concentration of the medium used to stress the cells is negligible, as the pellet was resuspended at least in 1000 volumes of low-salt buffer.

### SYTO9/propidium iodide assay by flow cytometry analysis

Flow cytometry was applied to follow multiple cellular parameters such as cell membrane integrity, in real time during low salt and recovery at high salt. Cells were centrifuged and the pellets were suspended in washing buffer (50 mM Tris-HCl pH 7.5; 70 mM KCl and 80 mM MgSO_4_) with a similar salt concentration to the one used during the cultivation (control or salt stress). The operation was done in triplicate for each point. Samples were stained with live stain (SYTO 9) during 15 minutes at 37 °C and the dead stain (propidium iodide: PI) was added just before analysis. Cells with damaged membranes will be penetrated by both stain SYTO 9 and PI. Flow cytometric analyses were carried out using the MACSQuant VYB (Miltenyi Biotec SAS, Bergisch Gladbach, Germany) equipped with three lasers, two scatter (FSC, SSC) and up to eight fluorescent channels. Green (SYTO 9) and red (PI) fluorescence, from cells excited by a 488 nm laser, was collected through a 525/50-nm or a 655–730-nm bandpass filter, respectively. A MACSQuantify software was used to assess the mortality rate in the different physiological conditions as described^15^. Manual gating of flow cytometry data was carried out on the basis of Pi and syto9 fluorescence intensity to discriminate PI+/Syto+ population from PI−/Syto− populations. Flow cytometry histogram profiles allow to differentiate Syto− population (low fluorescence yield) which are at least partly depleted in their DNA content from normal DNA content population (Syto+). Damaged or dead cells were determined from the ratio of PI+ (damaged membrane)/PI− (intact membrane).

### Measurement of *Hbt. salinarum* respiratory activity

The respiratory oxygen consumption was measured in *Hbt. salinarum*, an aerobic heterotroph. Archaea were exposed to various cultivated conditions as described in material and method (*H. salinarum* cells: cultivation and growth conditions). For each treatment, the rate of cellular respiratory activity was assayed in the same cell-suspension cultures before (salt shock) and after reactivation and in their corresponding supernatants. Air were bubble in 5 ml of a suspension of archaea until dissolved oxygen was in near equilibrium with the atmosphere, then cells were placed into a thermostated cell at 37 °C for respiration measurement. Oxygen consumption was quantified by using the Unisense oxygen microsensor. The respiratory data were normalized to 10^8^ cells. All experiments were done in triplicate.

### Microscopy

Time lapse microscopy was performed at the cell-imaging (M4D) platform of the IBS/ISBG on a temperature and illumination controlled IX81 inverted microscope using 100x (1.49NA) oil immersion objective and Dichroic Interference Contrast (DIC) imaging through diascopic LED light illumination (CoolLED™). Images were collected by a sCMOS Zyla camera (Andor), every 30 minutes as Z-stacks (PRIOR Piezo stage, one image every 0.5 µm; 5 µm range). Acquisitions were performed continuously for up to 96 hours and time/Z series were analyzed with ImageJ to select the best focal plane, and crop the region of interest from the acquired field of view, and further analyzed using Volocity (Quorum Technologies).

### Sample preparation for the neutron scattering experiments

Between 500 ml to 1.2 L of cultures were centrifuged at 4000 × g for 20 min. For the salt shock and recovery experiments, cell pastes were gently suspended in culture medium containing 2.5, 1.5 and 0.5 M NaCl and incubated for one hour or 24 hours at 37 °C with shaking. Culture of stressed cells were centrifuged at 6000 × g for 30 min. The culture was then divided in two for NS experiments: (a) a pellet used for the shocked cells and (b) a cell paste gently re-suspended in 4.2 M NaCl, incubated at 37 °C with shaking for one hour and centrifuged at 6000 g for 30 min, for the recovered cells. The shocked, recovered and control (4.2 M NaCl) pellets (400 mg each) were inserted in a 0.3-mm path length gold-coated aluminum flat sample older. Elastic neutron scattering (EINS) spectra were collected at the high resolution back scattering spectrometer IN13^38^ at the Institut Laue-Langevin (ILL), Grenoble, France. With an energy resolution of 8 μeV (full width at half maximum, FWHM) and an accessible momentum transfer range of 0.2 < Q < 4.9 Å^−1^, IN13 allows the investigation of molecular motions on a time scale up to 100 ps and with an amplitude from 1.3 Å to ~31 Å^37^. Data were acquired in the temperature range 278–315 K, above the freezing point of the water in order to avoid coherent scattering contributions arising from ice Bragg reflections. In order to optimize the signal to noise ratio, 4 hours of acquisition time per point were necessary. The program LAMP^39^ was used for data reduction, consisting in subtraction of the empty cell contribution and normalization with respect to a vanadium scan (a totally incoherent sample) to compensate for differences in detector efficiency and geometry. In order to avoid corrections from multiple scattering events, cell thickness and geometry were properly chosen to minimize neutron absorption from the sample (sample transmission ∼90%).

## Supplementary information

Supplementary video.

Supplementary information.

## Data Availability

All data needed to evaluate the conclusions in the paper are present in the paper and/or the Supplementary Materials. Data digital object identifier 10.5291/ILL-DATA.8-04-817.
