# Supplementary material for: Surviving salt fluctuations: stress and recovery in Halobacterium salinarum, an extreme halophilic Archaeon
Source: Sci Rep. 2020 Feb 24;10:3298. doi: 10.1038/s41598-020-59681-1 (PMC7040004; doi:10.1038/s41598-020-59681-1)
Supplement: Supplementary file 2 — Supplementary information. [file 41598_2020_59681_MOESM2_ESM.docx]

Surviving salt fluctuations: stress and recovery in *Halobacterium salinarum*, an extreme halophilic Archaeon

P. Vauclare,^1†^ F. Natali,^2,3†^ J. P. Kleman,^1^ G. Zaccai,^1,3^ B. Franzetti^1*^

Supplementary Materials

Fig. S1. Cell morphologies of *H. Salinarum* at various salt concentrations.

Video S1: Movie of time-lapse light microscopy of the *H. salinarum* morphological changes during recovery in 4.2M NaCl growth medium after a low-salt shock (1h) at 0.5M.

Fig. S2: Time-lapse light microscopy of the *H. salinarum* morphological changes.

Fig. S3: Q2 dependence of the normalized intensity from *H. salinarum* at 4.2 M NaCl.


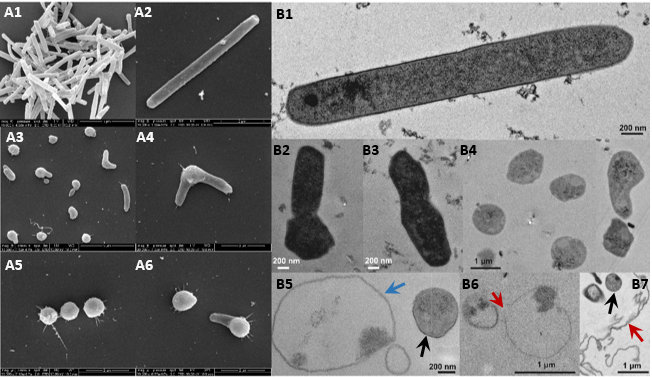


**Fig. S1. Cell morphologies of *H. Salinarum* at various salt concentrations.** Scanning electron microscopy (SEM) images (**A1-B6**) and transmitted electron microscopy (TEM, cryo-thin sections) images (**B1-B7**) of the corresponding representative morphologies feature of unstressed (**A1, A2** and **B1**) and low salt stressed *H. salinarum* (**B2** and **B3**, 2.5M; **A3-A6** and **B4**, 1.5M and **B5-B7**, 0.5M). TEM samples preparation: Cells were grown at 37°C in saline rich medium then centrifuged at 4500 x g for 5 min and prepared as described by (40). Digital images were obtained using a Tecnai G2 Spirit BioTwin microscope (FEI) operating at 120 kV with an Orius SC1000 CCD camera (Gatan). SEM samples preparation: Archaea were fixed in 2 % glutaraldehyde in saline TRIS buffer (50 mM, pH 7.5) with CaCl_2_ (1%, p/v) for 2 h at 4°C, washed overnight in the same buffer and attached to polylysine-coated glass coverslips. The archaea were dehydrated in a graded ethanol series and then desiccated in an EM CPD300 Leica critical point dryer. At last, they were coated with 6 nm platinum (Leica EM MED020) and visualized with a FEI Quanta 250 scanning electron microscope at an accelerating voltage of 5 KV. Black or blue arrow indicates intact cells with dense or empty cytosol, respectively. Red arrows indicate broken cells and membranes of lysed cells.


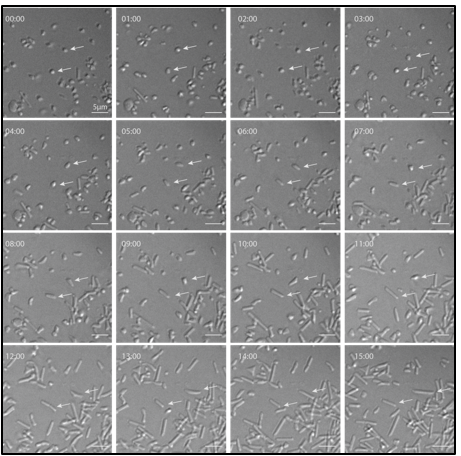


**Fig. S2: Time-lapse light microscopy.** *H. salinarum* morphological changes were monitored during the recovery in 4.2M NaCl growth medium from a low-salt shock (1h) at 2M NaCl. Cells were grown at 37°C in the microscopy chamber and observed by DIC on the confocal microscope. Image were collected every 30 minutes over a period of 15 hours. The recovery process starts from round-shape stressed cells which asynchronously generating small colonies with typical rode-shape cells after 8-15 hours of incubation in medium with 4.2M NaCl.


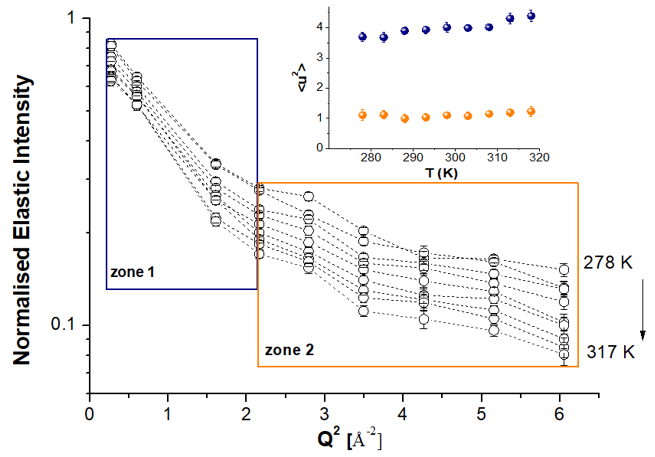


**Fig. S3:** **EINS data.** Q^2^ dependence of the normalized intensity from *H. salinarum* at 4.2 M NaCl measured at different temperatures from 278 to 317K top-down. The MSD versus temperature are shown in the inset. Orange and blue symbols refer to the two Q^2^ zones (blue = zone 1, orange = zone 2) highlighted in the figure.

Due to relatively long acquisition times to favor good signal to noise data (4 h/point), a check of sample stability during the measuring time was performed. Short scans (16 of 15 minutes each per temperature point) were continuously acquired and compared. From the slope of the semi-logarithmic plot of the incoherent scattering function we extracted the Mean Square Displacements (MSD) for a given T (37). For the sake of completeness the MSD were calculated in two different Q ranges corresponding to large and small amplitudes motions, respectively. The MSD versus T, reported in the inset of fig. S6, show almost no temperature dependence for the small amplitude motions (orange) in agreement with previous findings (13). Thus, we focused on the temperature sensitive large amplitude fluctuations.

An effective average force constant for sample dynamics, < k >, can be calculated from the slope of <u^2^> as a function of temperature, by applying a quasi-harmonic approximation (41):

<k^'>=0.00276/((d<u^2>)⁄dT)

Additional references

40) Jacq, M. et al. The cell wall hydrolase Pmp23 is important for assembly and stability of the division ring in Streptococcus pneumoniae. Sci. Rep. 8, 7591 (2018).

41) Zaccai, G. How soft is a protein? A protein dynamics force constant measured by neutron scattering. Science 288, 1604-1607 (2000).
